# Associated factors influencing pain and slower gait speed in older adults with knee osteoarthritis: a cross-sectional study

**DOI:** 10.3389/fmed.2026.1915220

**Published:** 2026-08-07

**Authors:** Yu Qu, Peipei Wang, Hualong Xie, Jiaqi Yang, Jialin Fan, Xin Zhang, Ming Huo

**Affiliations:** 1 Qilu Hospital of Shandong University, Jinan, China; 2 School of Rehabilitation Science and Engineering, University of Health and Rehabilitation Sciences, Qingdao, Shandong, China; 3 Department of Rehabilitation Medicine, Qingdao Central Hospital, University of Health and Rehabilitation Sciences (Qingdao Central Medical Group), Qingdao, Shandong, China; 4 Wangjing Hospital, China Academy of Chinese Medical Sciences, Beijing, China; 5 Beijing Chaoyang District Sanhuan Cancer Hospital, Beijing, China; 6 Shandong Key Laboratory of Neurorehabilitation, University of Health and Rehabilitation Sciences, Qingdao, China; 7 Jilin Province Power Hospital, Changchun City, Jilin, China; 8 Xi'an Electric Power Central Hospital, Xi'an City, Shaanxi, China

**Keywords:** associated factor, cross-sectional study, gait speed, knee osteoarthritis, older adults, pain

## Abstract

**Objective:**

Given the complex etiology and unclear pathogenesis of knee osteoarthritis (KOA), its prevention is important. This study aimed to investigate factors independently associated with pain severity and slower gait speed in older adults with KOA.

**Design:**

This cross-sectional study was performed at a Rehabilitation Training Center. In total, 120 patients at our hospital between 2024 and 2025 with a confirmed diagnosis of KOA and 119 healthy individuals were matched for demographic characteristics. Ultrasound-derived rectus femoris (RF) parameters—muscle thickness and cross-sectional area (CSA)—were measured at rest and during contraction. Pain was assessed using the Visual Analog Scale (VAS). Additional measures included knee flexion range of motion (ROM), knee joint position sense (proprioception), and the 10-meter walk test (10MWT) for gait speed.

**Results:**

Compared with healthy controls, KOA patients exhibited significantly lower walking speed, knee ROM, proprioceptive accuracy, RF muscle thickness and CSA, and tibial rotation angles. In KOA, faster gait speed (shorter 10MWT time) was positively correlated with knee ROM, RF thickness and CSA (both at rest and during contraction), and tibial rotation angles, while it was negatively correlated with VAS score and age. Multivariable regression analyses identified age, pain, RF thickness at rest, and total tibial rotation as independent correlates of slower gait speed. Additionally, higher body mass index, female sex, and slower gait speed were independently associated with greater knee pain.

**Conclusions:**

Older age, greater pain, reduced resting RF thickness, and diminished total tibial rotation were independently associated with slower gait speed; higher BMI, female sex, and slower gait speed were independently linked to more severe knee pain. These associations should be confirmed in future prospective interventional studies.

## Introduction

Knee osteoarthritis (KOA) is a chronic, whole-joint disorder that affects articular cartilage, subchondral bone, synovium, menisci, ligaments, and periarticular tissues—rather than being confined to cartilage alone. Its pathogenesis is intricately linked to mechanical, inflammatory, and metabolic disturbances that disrupt normal joint homeostasis (1–3). Patients typically present with pain, functional limitation, and joint deformity (4). In 2020, KOA affected nearly 365 million people globally, and this number is projected to rise by 74.9% by 2050, imposing a substantial symptom burden and significant socioeconomic costs (5–7).

Established associated factors include higher BMI, advancing age, female sex, knee malalignment, repetitive joint stress, quadriceps weakness, and genetics predisposition (8, 9), Despite this knowledge, no disease-modifying therapy currently exists, making the identification of modifiable functional determinants a research priority (10–13).

In healthy joints, the tibia rotates externally during extension and internally during flexion-a phenomenon known as the screw-home movement (SHM), which plays a critical role in knee stability at terminal extension (14, 15). However, In KOA, cartilage degradation, osteophyte formation, and capsular laxity disrupt this coupling, which can lead to dysfunction and deformity. As severity progresses, the range of tibial rotational motion decreases, destabilizing the joint during weight-bearing activities (16). Moreover, reduced SHM in osteoarthritic knees increases stress on articular cartilage and accelerates its loss (14). Previous studies have primarily assessed tibial rotation in static sitting positions, which does not adequately reflect dynamic conditions. In the present study, we therefore employed a three-dimensional (3D) gait analysis system to quantify tibial rotation during walking. Although pathological structural changes are considered major contributors to knee pain, this relationship requires further empirical support.

KOA commonly results in walking difficulties and reduced gait speed-a simple yet robust indicator of overall health and wellbeing in older adults, and a powerful predictor of mortality (17, 18). Pain may further alter biomechanics through compensatory gait adaptations-such as toe-in gait, trunk leaning, or reduced walking speed-that modify knee loading (19, 20).

Ultrasonography has emerged as a fast, safe, and portable tool for assessing muscle morphological parameters, including thickness and cross-sectional area, with reliability and validity comparable to magnetic resonance imaging and computed tomography (21). In adults with KOA, ultrasound demonstrates high inter-rater reliability for lower-limb muscle assessment, correlates negatively with patient-reported functional scores, and may even outperform hand-held dynamometry in certain contexts (22). In addition, impaired knee joint proprioception can substantially affect somatosensory and motor control around the joint (23, 24), contributing to functional limitation, disability, and increased fall risk in individuals with KOA (25).

KOA is driven by a multifactorial etiology involving mechanical overload, low-grade synovial inflammation, and metabolic disturbances-all of which contribute to quadriceps muscle weakness and atrophy. This muscle dysfunction is a key determinant of joint instability, pain exacerbation, and gait abnormalities. Given its central role in disease progression, identifying modifiable factors associated with pain and slower gait speed is of clinical importance.

Specifically, this study aimed to: (1) compare ultrasound-derived muscle morphological parameters-rectus femoris (RF) thickness and cross-sectional area (CSA) and functional outcomes-pain, knee range of motion (ROM), joint position sense, and gait speed between KOA patients and demographically matched healthy controls; and (2) identify factors independently associated with pain severity and reduced gait speed in adults with KOA. We hypothesized that reduced RF thickness, smaller CSA, and diminished tibial rotation would be significantly associated with greater pain and slower gait speed, after adjusting for potential confounders including age, sex, and body mass index (BMI).

Despite the established roles of quadriceps dysfunction and altered knee kinematics in KOA progression, several knowledge gaps remain. First, most previous studies have examined muscle morphology and dynamic joint kinematics in isolation, leaving their independent and combined associations with gait speed unexplored. Second, although pain is known to correlate with reduced gait speed, whether this association is mediated by muscle atrophy or rotational deficits-or whether these factors exert independent effects-has not been determined within a unified multivariate framework. Third, tibial rotational abnormalities in KOA have been primarily described using static imaging or silico modeling studies; dynamic rotational excursion during overground walking-the condition most relevant to patients' daily function-remains poorly quantified. This study was designed to address these gaps by simultaneously evaluating ultrasound-derived RF morphology, 3D gait-derived tibial rotational kinematics, pain, and gait speed in a well-characterized cohort of older adults with KOA.

## Materials and methods

### Study design, setting, and participants

Participants were recruited through outpatient clinics and community-based health promotion activities associated with Beijing Chaoyang District Sanhuan Cancer Hospital. All participants who met the eligibility criteria during the study period (2024–2025) were consecutively enrolled to minimize selection bias.

The diagnostic criteria for primary KOA included the following: (1) the patient had experienced recurrent knee pain in the past month; (2) patients were aged ≥50 years; (3) morning stiffness lasted no more than 30 min; (4) bone fricative or bone rubbing sensation was present during movement; (5) a radiograph taken in the standing or weight-bearing position revealed narrowing of the joint space, sclerosis of the subchondral bone, cystic degeneration, or osteophyte formation at the joint edge; and (6) MRI showed cartilage injury, osteophyte formation, subchondral bone marrow edema and/or cystic changes, degenerative tear of the meniscus, and partial or complete loss of cartilage. A diagnosis of KOA can be confirmed if criteria (1) and (2); (3), (4), (1), and (5); or (1) and (6) are satisfied (26). For participants with bilateral KOA, we intentionally selected the more symptomatic/radiographically severe knee for inclusion in our analysis.

KOA inclusion criteria were as follows: (1) the patient meets the diagnostic criteria for primary KOA; (2) patients aged ≥50 years; and (3) those capable of providing conscious and voluntary consent for study participation. We screened healthy controls by history (no prior knee pain, surgery/trauma, or pain on squatting/stair climbing) and physical exam (no tenderness, effusion, or crepitus). The control group comprised healthy individuals matched for age and BMI. The inclusion criteria for healthy controls were: (1) No previous knee pain (2) Absence of musculoskeletal or central nervous system disorders; (3) Community-dwelling and capable of self-care.

The KOA exclusion criteria were the presence of (1) cognitive impairment or neurological dysfunction, (2) rheumatoid arthritis or osteonecrosis, (3) difficulty walking, (4) history of knee joint trauma or knee surgery, (5) foot and ankle deformities, and (6) systemic or knee malignant tumors in both groups. (7) severe cardiovascular disease (e.g., unstable angina, severe heart failure, or uncontrolled hypertension) or severe respiratory disease that could affect exercise capacity; and (8) history of hip or lumbar spine surgery that could alter gait biomechanics. The exclusion criteria for healthy controls were: (1) With multiple underlying diseases and being physically frail; (2) Inability to complete the planned exercise protocol in the study.

### Experimental methods

Knee joint ROM, knee joint position sense, rectus femoris muscle thickness, cross-sectional area and tibial rotation angle during walking were assessed.

### Ultrasound

Measurements were obtained with the participant in the supine position and the tested lower limb fully extended (0° hip and knee extension). Using an ultrasound device (Sonosite M-Turbo, Japan), the muscle thickness and CSA of the RF were measured 10 cm proximal to the patella during rest or contraction. Both legs were measured. The probe was placed perpendicular to the longitudinal axis of the RF with minimal pressure on the thigh soft tissue. Muscle thickness and cross-sectional area were measured in twice to calculate means for further analysis. All measurements of muscle thickness and cross-sectional area were performed by a single investigator that the investigator was blinded to participants' clinical characteristics, grouping, and study hypotheses. All ultrasound images were obtained by a physician with 5 years of experience in musculoskeletal ultrasound imaging (27).

### Pain severity

Knee pain during activities of daily living (ADLs) was measured using a Visual Analog Scale (VAS) (score range, 0–10 points). The patients were asked to mark their subjective pain levels on a 10-cm scale. Accordingly, the value 0 indicates that there is no pain, and the value 10 indicates the most severe pain. The distance between the baseline and the marked point is recorded as centimeters (28).

### Knee joint flexion ROM

For the active knee ROM flexion measurement, the participants lay supine, and the axis of a goniometer was placed at the intersection of the thigh and shank at the knee joint. The fixed arm of the goniometer was placed on a line joining the greater trochanter with the lateral femoral condyle; the center was placed on the lateral femoral condyle and the free arm was placed on a line joining the fibular head and lateral malleolus. The value retained was obtained by subtracting the measurement with the knee flexed from the one with the hip extended on the table (29). Participants were instructed to actively flex the knee as far as possible, and the maximum flexion angle was recorded. All measurements were performed by a single trained physical therapist with over 5 years of clinical experience, who was blinded to participants'group allocation. Each measurement was performed twice, and the mean value was used for analysis.

### 3D gait analysis

In contrast to patient-reported outcome measures, which are subject to individual biases and can be limited in their ability to provide an objective assessment of joint function, 3D motion analysis provides an objective, quantitative, reproducible, and standardized assessment of joint kinematics (30).

A Noraxon MR4.0 biomechanics system (Scottsdale, AZ, USA) synchronized and recorded the data. Seven sensors were placed on the dorsal foot, fastened to the middle of the thigh and middle of the calf, and in the sacral region of each participant with bandages, with the participant in a neutral standing position. Calibration was performed with participants in the upright position to determine the value of the 0° in the joints (31). We measured the tibia internal/external rotation angle during 30-s walking along a straight line. The walk was performed in a straight line of at least 10 m, and the participants performed at least four repetitions to obtain two complete and correct walks for analysis. The maximum (maximum range) and minimum (minimum range) joint angles and ROM of the knee were calculated for all gait phases. In addition, operator practiced operating the Noraxon MR4.0 biomechanics system under the guidance of a Noraxon technician for approximately 1 month. All the datas were collected by the same operator that the operator was blinded to participants'clinical characteristics, grouping, and study hypotheses.

### 10MWT

The 10-m walk test (10 MWT) involved timing (in seconds) the middle 10 m of a 15-m walkway under a dynamic start condition (i.e., timing from first foot crossing of the 2-m mark to first foot crossing of the 12-m mark, with continued walking to the 15-m mark), as previously reported (32). Thus, a higher time in seconds denotes a slower walking speed, and lower values indicate faster gait. In this study, 10MWT results are reported in meters per second (m/s). Timing was measured using a smartphone stopwatch operated by a single trained investigator. Each measurement was repeated twice, and the mean value was considered in subsequent analysis.

### Knee position sense

To evaluate knee joint position sense, joint position matching tasks were performed by participants in the prone position on a massage table while blindfolded. The participants were asked to actively match the positions with their indicator limb, while the experimenter moved the reference limb to the tested angles (from 40–70°of knee flexion) using a digital inclinometer. All measurements were performed by a single trained physical therapist who was blinded to participants' group allocation. The mean of three measurements was recorded for both limbs (33).

### Bias control

To address potential sources of bias, we consecutively enrolled all eligible patients from 2024 to 2025. All neurological assessments were performed by the same investigator (blinded to group allocation) to ensure consistency. The study protocol was pre-registered and approved by the Chinese Clinical Trial Registry (ChiCTR2400086711).

### Sample size

In this study, A total of 10 factors were used as a basis, including such as age, sex, BMI, VAS, active knee ROM, Knee joint position sense, 10MWT, RF muscle thickness at rest/during contraction, Knee total rotation. (Some variables were highly correlated. To avoid multicollinearity, we retained only the most independent indicators. We removed “knee internal rotation” and “knee external rotation” and kept only total knee rotation. We removed RF CSA (cross-sectional area) and kept only RF thickness.) *A priori* sample size estimation was performed using GPower 3.1.9.7 for linear multiple regression (fixed model, *R*^2^ deviation from zero). Based on 10 candidate predictor variables, a medium effect size (*f*^2^ = 0.15), a significance level of α = 0.05, and a statistical power of 1-β = 0.80, the required total sample size was calculated as 118 participants. KOA group: 120 participants were enrolled (≥118); Healthy control group: 119 participants were enrolled (≥118).

### Statistical analysis

All continuous variables were retained in their original form and entered as continuous predictors in the regression models, without data-driven dichotomization, to preserve statistical power and avoid information loss. Missing data were minimal (< 5% for all variables), therefore, we performed complete case analysis, excluding participants with any missing outcome data. The Shapiro–Wilk test was used to assess the normality of continuous variable distributions, and the Levene test was used to evaluate the homogeneity of variances between groups, to guide the selection of appropriate parametric or nonparametric tests for group comparisons. Comparisons of outcome variables between the two groups were performed using Student *t*-test for parametric continuous variables and the Mann–Whitney *U*-test for nonparametric continuous variables (29). Cohen *d* was used to estimate the effect size, with value 0.2 defined as a small effect size, >0.5 as a medium effect size, and >0.8 as a large effect size. The Spearman rank correlation coefficient (rho) was used to analyze correlations between the variables assessed in patients with KOA. Variables with *p* < 0.10 in univariate analyses were considered for entry into the multivariable models. The following method was used: “Linear multiple regression”: fixed model, *R*^2^ deviation from zero. Multiple linear regression analysis was performed with stepwise selection (Stepwise method) for variable entry, using a probability of *F*-entry ≤ 0.05 and *F*-removal ≥ 0.10. The absence of problematic multicollinearity in the final model (all VIFs < 2) confirms that the retained variables do not introduce collinearity issues. Statistical significance was set at *p*-value < 0.05, and data were analyzed using SPSS software (version 27.0, IBM Corp., Armonk, NY, USA) and Graph Pad Prism 8 (San Diego, CA, USA).

### Ethics statements

This study was approved by Ethics Committee of Beijing Chaoyang District Sanhuan Cancer Hospital and was conducted in accordance with local legislation and institutional requirements. All the participants provided written informed consent for participation.

## Results

The demographic, clinical, and ultrasound characteristics of the study population are summarized in Table 1. A total of 239 participants were enrolled, comprising 120 patients with KOA and 119 healthy controls. One KOA patient had missing ultrasound thickness and area data; this case was excluded listwise from all regression analyses, yielding a final effective sample size of 119 for the multivariable models. The average age of KOA participants was 64.97 ± 0.9 years, with a mean BMI of 25.93 ± 0.1 kg/m^2^; 102 (85%) were female and 18 (15%) were male. The index side was left in 50 participants (41.7%) and right in 70 (58.3%). The average age of healthy individuals was 63.47 ± 0.9 years, with a mean BMI of 25.33 ± 0.9 kg/m^2^; it included 98 females (82.4%) and 21 males (17.6%), with the index side being left in 51 participants (42.9%) and right in 68 (57.1%).

**Table 1:** Demographic characteristics of the patients with knee osteoarthritis and healthy individuals.

Variable	Patients with KOA (*n* = 120) *N* (%)/Mean ±SD	Healthy individuals (*n* = 119) *N* (%)/Mean ±SD	*p*-value	Cohen *d* between groups
Age (years)	64.97 ± 0.9	63.47 ± 0.9	0.156	0.184
Height (cm)	161.47 ± 0.0	161.56 ± 0.8	0.929	−0.011
Weight (kg)	67.81 ± 0.7	66.21 ± 2.4	0.300	0.134
BMI (kg/m^2^)	25.93 ± 0.1	25.33 ± 0.9	0.156	0.184
Sex				
Male	18 (15)	21 (17.6)	0.580	0.036
Female	102 (85)	98 (82.4)		
Index side				
Left	50 (41.7)	51 (42.9)	0.852	0.012
Right	70 (58.3)	68 (57.1)		

KOA, knee osteoarthritis; SD, standard deviation; BMI, body mass index.

As shown in Table 2, patients with KOA reported significantly greater knee pain than controls (*p* < 0.001), with a large effect size (Cohen's *d* ≥ 1.0). Knee flexion ROM was significantly reduced in the KOA group (*p* < 0.001, medium effect size, Cohen's *d* ≥ 0.5), and joint position sense (proprioception) was also poorer (*p* = 0.001, small effect size, Cohen's *d* < 0.5). The 10-m walking time was relatively slower in the KOA group (*p* < 0.001, Cohen *d* = 0.527); their walking speed was 1.150 ± 0.18 m/s, while the age-, sex-, and BMI-matched healthy controls walked at 1.240 ± 0.16 m/s. This 0.7-s difference corresponds to a gait speed reduction of about 0.08 m/s. The difference was statistically significant (*p* < 0.001) with a moderate effect size (Cohen's *d* = −0.496). RF muscle thickness was modestly smaller in the KOA group both at rest (*p* = 0.012, Cohen *d* = −0.330) and during isometric contraction (*p* = 0.003, Cohen *d* = −0.391). Similarly, RF CSA was also modestly reduced at rest (*p* = 0.001, Cohen *d* = −0.417) and during contraction (*p* < 0.001, *d* = −0.439). During walking, the KOA patient group exhibited significantly smaller angles of tibial internal rotation (*p* = 0.009, Cohen *d* = −0.339), external rotation (*p* < 0.001, Cohen *d* = −1.030), and total rotationa (*p* < 0.001, Cohen *d* = −1.067) compared with controls with large effect sizes for external and total rotation and a small-to-moderate effect for internal rotation.

**Table 2:** Clinical and ultrasound parameters (rest/contraction) in patients with knee osteoarthritis and healthy individuals.

Variable	Patients with KOA (*n* = 120)	Healthy individuals (*n* = 119)	*p*-value	Cohen *d* between groups	95% CI
VAS score	3.21 ± 0.8	0	< 0.001	1.438	1.152,1.721
Knee ROM (°)	128.31 ± 5.0	137.69 ± 0.4	< 0.001	−0.742	−1.004, −0.479
Knee joint position sense (°)	7.14 ± 0.6	5.43 ± 0.1	0.001	0.430	0.173,0.686
10 MWT result (s)	8.91 ± 0.5	8.21 ± 0.1	< 0.001	0.527	0.268, 0.784
10 MWT result (m/s)	1.150 ± 0.18	1.240 ± 0.16	< 0.001	−0.496	−0.753, −0.238
RF muscle thickness at rest (mm)	9.53 ± 0.3	10.74 ± 0.0	0.012	−0.330	−0.585, −0.074
RF CSA at rest (cm^2^)	2.50 ± 0.9	2.90 ± 0.9	0.001	−0.417	−0.673, −0.160
RF muscle thickness during contraction (mm)	13.94 ± 0.7	15.64 ± 0.3	0.003	−0.391	−0.647. −0.134
RF CSA during contraction (cm^2^)	2.81 ± 0.1	3.31 ± 0.1	< 0.001	−0.439	−0.696, −0.182
Knee internal rotation (°)	8.43 ± 0.1	9.22 ± 0.1	0.009	−0.339	−0.594, −0.083
Knee external rotation (°)	8.12 ± 0.7	10.92 ± 0.6	< 0.001	−1.030	−1.299, −0.759
Total knee rotation (°)	16.63 ± 0.5	20.12 ± 0.9	< 0.001	−1.067	−1.337, −0.795

Values are displayed as mean ± SD. SD, standard deviation; CI: confidence intervals; VAS, visual analog scale; ROM, range of motion; 10 MWT, 10-m walk test; RF, rectus femoris; CSA, cross-sectional area.

Univariate correlation analyses (Table 3) revealed that in KOA patients, faster walking speed (shorter 10MWT time) was positively associated with greater knee flexion ROM (*r* = 0.338, *p* < 0.001), RF muscle thickness at rest (*r* = 0.305, *p* < 0.001), RF CSA at rest (*r* = 0.235, *p* = 0.010), RF muscle thickness during contraction (*r* = 0.219, *p* = 0.017), RF CSA during contraction (*r* = 0.194, *p* = 0.034), knee internal rotation (*r* = 0.211, *p* = 0.021), knee external rotation (*r* = 0.216, *p* = 0.018), and total knee rotation (*r* = 0.317, *p* < 0.001). Conversely, older age (*r* = −0.351, *p* < 0.001) and higher VAS pain score (*r* = −0.278, *p* = 0.002) were negatively associated with the walking speed. However, sex (*r* = −0.038, *p* = 0.681), BMI (*r* = 0.051, *p* = 0.580), and knee joint position sense (*r* = −0.041, *p* = 0.656) showed no significant correlation with 10MWT.

**Table 3:** Correlation matrix of demographic, clinical, and biomechanical variables with VAS pain and 10MWT gait speed in KOA patients (*n* = 120).

Domain	Variable	Patients with KOA (*n* = 120)	*r* (VAS score)	*p*-value	*r* (10MWT (m/s))	*p*-value
Demographic & clinical	VAS	3.21 ± 0.8			−0.278	0.002
	10MWT (m/s)	1.150 ± 0.18	−0.278	0.002		
	Sex	Female: 102 (85)	0.193	0.034	−0.038	0.681
	Age (years)	64.97 ± 0.9	0.068	0.461	−0.351	< 0.001
	BMI (kg/m^2^)	25.93 ± 0.1	0.167	0.068	0.051	0.580
	Knee ROM (°)	128.31 ± 5.0	−0.159	0.083	0.338	< 0.001
	Knee joint position sense (°)	7.14 ± 0.6	0.013	0.891	−0.041	0.656
Muscle ultrasound	RF muscle thickness at rest (mm)	9.53 ± 0.3	0.003	0.978	0.305	< 0.001
	RF CSA at rest (cm^2^)	2.50 ± 0.9	−0.051	0.581	0.235	0.010
	RF muscle thickness during contraction (mm)	13.94 ± 0.7	−0.008	0.386	0.219	0.017
	RF CSA during contraction (cm^2^)	2.81 ± 0.1	−0.094	0.311	0.194	0.034
Knee rotation	Knee internal rotation (°)	8.43 ± 0.1	−0.208	0.023	0.211	0.021
	Knee external rotation (°)	8.12 ± 0.7	0.020	0.829	0.216	0.018
	Total knee rotation (°)	16.63 ± 0.5	−0.217	0.017	0.371	< 0.001

BMI, body mass index; VAS, Visual Analog Scale; ROM, range of motion; 10 MWT, 10-m walk test; RF, rectus femoris; CSA, cross-sectional area.

For pain outcomes in KOA patients, univariate analysis indicated that female sex was positively associated with the VAS score (*r* = 0.193, *p* = 0.034), whereas knee internal rotation (*r* = −0.208, *p* = 0.023), total knee rotation (*r* = −0.217, *p* = 0.017), and walking speed (*r* = −0.278, *p* = 0.002) were negatively associated with pain. No significant correlations with VAS were found for age (*r* = 0.068, *p* = 0.461), BMI (*r* = 0.167, *p* = 0.068), knee ROM (*r* = −0.159, *p* = 0.083), knee joint position sense (*r* = 0.013, *p* = 0.891), and all ultrasound-derived muscle parameters (all *p* > 0.05; Table 3).

All 10 independent variables were entered into the multiple linear regression model for walking speed in KOA patients. Preliminary assumption checks were satisfied: the scatterplot of residuals vs. predicted values showed a random distribution around zero with no obvious curvilinear pattern, and no multicollinearity was detected (all VIFs < 2). The Durbin–Watson statistic of 1.978 fell within the acceptable range (1.5–2.5), indicating no serious autocorrelation. The multivariate linear regression model for 10MWT (*F*_41, 141_ = 0.440, *p* < 0.001), and explained 26.8% of the variance (*R*^2^0 = 0.268, adjusted *R*^2^0 = 0.242). As displayed in Table 4. In the exploratory correlation analyses, we observed that independent associations with slower walking speed were observed for older age (Beta = −0.218, 95 % CI [−0.009, −0.001], *p* = 0.013), smaller total knee rotation (Beta = 0.194, 95 % CI [0.001, 0.019], *p* = 0.027), reduced RF muscle thickness at rest (Beta = 0.199, 95 % CI [0.002, 0.024], *p* = 0.021), and higher VAS pain score (Beta = −0.224, 95 % CI [−0.036, −0.006], *p* = 0.007).

**Table 4:** Multivariate linear regression model of the 10MWT (m/s) in patients with knee osteoarthritis (Stepwise method, effective *n* = 119).

Variable	B	SE	Beta	t	*p*-value	95% CI		*R* ^2^	adjusted *R*^2^	*F* (*p*)	VIF	Durbin–Watson
Constant	1.250	0.179		6.992	< 0.001	0.894	1.604	0.268	0.242	10.440 (< 0.001)		1.978
Total knee rotation (°)	0.010	0.004	0.194	2.233	0.027	0.001	0.019				1.171	
Age (years)	−0.005	0.002	−0.218	−2.526	0.013	−0.009	−0.001				1.162	
VAS score	−0.023	0.008	−0.224	−2.725	0.007	−0.039	−0.006				1.057	
RF muscle thickness at rest (mm)	0.013	0.005	0.199	2.350	0.021	0.002	0.024				1.1123	

VAS, visual analog scale; RF, rectus femoris; CI, confidence interval; VIF, variance inflation factor; SE, standard error.

The multivariable regression model for pain severity (VAS) in KOA patients is shown in Table 5. The model was significant (*F*_31, 157_ = 0.640, *p* < 0.001), accounting for 16.6% of the variance (*R*^2^0 = 0.166, adjusted *R*^2^0 = 0.144) in patients with KOA was shown in Table 5. The Durbin–Watson statistic was 1.608, all VIFs were < 2, and residual plots met linearity and homoscedasticity assumptions. In the exploratory correlation analyses, we observed that higher BMI (Beta = 0.235, 95 % CI [0.034, 0.223], *p* = 0.008), female sex (Beta = 0.237, 95 % CI [0.314, 2.005], *p* = 0.008), and slower gait speed (Beta = −0.286, 95 % CI [−4.507, −1.163], *p* = 0.001) were independently associated with greater knee pain.

**Table 5:** Multivariate linear regression model of the VAS score in patients with knee osteoarthritis (Stepwise method, effective *n* = 119).

Variable	B	SE	Beta	t	*p*-value	95% CI		*R* ^2^	adjusted *R*^2^	*F* (*p*)	VIF	Durbin–Watson
Constant	2.153	1.661		1.296	0.197	−1.137	5.443	0.166	0.144	7.640 (< 0.001)		1.608
10 MWT result (m/s)	−2.835	0.844	−0.286	−3.359	0.001	−4.507	−1.163				1.003	
BMI	0.128	0.048	0.235	2.683	0.008	0.034	0.223				1.055	
Sex	1.159	0.427	0.237	2.715	0.008	0.314	2.005				1.053	

BMI, body mass index; 10 MWT, 10-m walk test; CI, confidence interval; VIF, variance inflation factor; SE, standard error.

Among healthy controls (Table 6), univariate correlations with walking speed (10MWT-m/s) were positive for total knee rotation (*r* = 0.260, *p* = 0.004), RF muscle thickness during contraction (*r* = 0.250, *p* = 0.006), RF CSA during contraction (*r* = 0.188, *p* = 0.041), and knee flexion ROM (*r* = 0.200, *p* = 0.029). Negative correlations were observed for age (*r* = −0.233, *p* = 0.011) and knee joint position sense (*r* = −0.235, *p* = 0.010). No significant associations were found with sex (*r* = −0.066, *p* = 0.476), BMI (*r* = 0.028, *p* = 0.759), RF muscle thickness at rest (*r* = 0.145, *p* = 0.116), RF CSA at rest (*r* = 0.129, *p* = 0.160), knee internal rotation (*r* = 0.167, *p* = 0.069), and knee external rotation (*r* = 0.163, *p* = 0.076).

**Table 6:** Correlation matrix of demographic, clinical, and biomechanical variables with 10MWT gait speed in healthy individuals (*n* = 119).

Domain	Variable	Healthy individuals (*n* = 119)	*R* (10MWT [m/s])	*p*-value
Demographic & clinical	10MWT (m/s)	1.240 ± 0.16		
	Sex	Female 98 (82.4)	−0.066	0.476
	Age (years)	63.47 ± 0.9	−0.233	0.011
	BMI (kg/m^2^)	25.33 ± 0.9	0.028	0.759
	Knee ROM (°)	137.69 ± 0.4	0.200	0.029
	Knee joint position sense (°)	5.43 ± 0.1	−0.235	0.010
Muscle ultrasound	RF muscle thickness at rest (mm)	10.74 ± 0.0	0.145	0.116
	RF CSA at rest (cm^2^)	2.90 ± 0.9	0.129	0.160
	RF muscle thickness during contraction (mm)	15.64 ± 0.3	0.250	0.006
	RF CSA during contraction (cm^2^)	3.31 ± 0.1	0.188	0.041
Knee rotation	Knee internal rotation (°)	9.22 ± 0.1	0.167	0.069
	Knee external rotation (°)	10.92 ± 0.6	0.163	0.076
	Total knee rotation (°)	20.12 ± 0.9	0.260	0.004

BMI, body mass index; ROM, range of motion; 10 MWT, 10-m walk test; RF, rectus femoris; CSA, cross-sectional area.

Multivariable analysis for walking speed in the healthy control group (Table 7) yielded a significant model (*F*_41, 147_ = 0.208, *p* < 0.001, *R*^2^0 = 0.202, adjusted *R*^2^0 = 0.174). The Durbin–Watson statistic was 2.099, VIFs were < 2, and residual plots confirmed model assumptions. In the exploratory correlation analyses, we observed that independent predictors of slower walking speed included smaller RF thickness during contraction (Beta = 0.263, 95 % CI [0.003, 0.016], *p* = 0.003), worse ROM (Beta = 0.209, 95 % CI [0.001, 0.006], *p* = 0.016), older age (Beta = −0.193, 95 % CI [−0.007, 0.001], *p* = 0.024) and poorer knee position sense (Beta = −0.210, 95 % CI [−0.019, −0.002], *p* = 0.015).

**Table 7:** Multivariate linear regression model of the 10MWT (m/s) result in healthy individuals (Stepwise method, effective *n* = 119).

Variable	B	SE	Beta	t	*p*-value	95% CI		*R* ^2^	adjusted *R*^2^	*F* (*p*)	VIF	Durbin–Watson
Constant	0.908	0.242		3.756	< 0.001	0.429	1.387	0.202	0.174	7.208 (< 0.001)		2.099
RF muscle thickness during contraction (mm)	0.010	0.003	0.263	3.082	0.003	0.003	0.016				1.041	
ROM (°)	0.003	0.001	0.209	2.446	0.016	0.001	0.006				1.043	
Age (years)	−0.004	0.002	−0.193	−2.287	0.024	−0.007	−0.001				1.020	
Knee joint position sense (°)	−0.011	0.004	−0.210	−2.475	0.015	−0.019	−0.002				1.025	

RF, rectus femoris; CI, confidence interval; VIF, variance inflation factor; SE, Standard error.

## Discussion

This cross-sectional study explores a relatively under-investigated area into the multifactorial risk profiles associated with pain severity and walking speed decline in older adults with KOA. Our findings provides additional evidence that patients with KOA exhibit significantly poorer outcomes across multiple domains, including walking speed, knee ROM, proprioceptive accuracy, RF morphology (muscle thickness and CSA), and dynamic tibial internal and external rotations, than demographically matched healthy controls. Notably, multivariate regression analyses identified distinct sets of independent associated factors of slower gait speed and greater knee pain. To our knowledge, few studies have simultaneously examined evaluate ultrasound-derived RF morphology and dynamic tibial rotational kinematics within a multivariate model to determine their independent contributions to gait speed and pain in this population.

### Clinically meaningful gait speed reduction

Our findings extend the evidence that individuals with symptomatic KOA have an approximately 9-fold higher risk of a fast decline trajectory in gait speed compared with those without radiographic OA or knee pain (18). In our cohort, slower gait speed was independently associated with older age, greater pain, reduced rectus femoris thickness, and diminished total tibial rotation. Pain likely contributes to gait slowing through compensatory biomechanical adaptations that reduce joint loading but promote disuse atrophy. Indeed, KOA patients often experience reduced lower limb strength due to pain-related physical inactivity (34), and weakening of the quadriceps and hamstrings has been linked to decreased walking stability (35). Diminished tibial rotation indicates altered knee kinematics, including disruption of the normal “screw-home” mechanism, which exacerbates mediolateral load imbalance and may accelerate cartilage damage while compromising knee stability during extension (14).

Perera et al. (36) proposed that best initial estimates for meaningful change in gait speed are ~0.05 m/s for small change and ~0.10 m/s for substantial change. In our study, the 0.7-s difference in the 10-meter walk test translates to a gait speed reduction of approximately 0.09 m/s, approaching the threshold for substantial clinical relevance. This suggests that KOA patients in our cohort exhibit a clinically relevant reduction in walking speed compared with their healthy peers.

### Role of rectus femoris morphology

The knee extensor (KE) mechanism, primarily composed of the quadriceps femoris, is crucial for joint stability and load attenuation during walking (37). As a key component of this muscle complex, the RF serves as one of the most important dynamic stabilizers of the knee. Impairments in KE muscle function in KOA may increase the functional demand on the lower limb muscles during activities of daily living, contributing to to disability, reduced gait speed and greater fall risk (38). In our study, RF muscle thickness and CSA at rest/during contraction were positively correlated with walking speed in patients with KOA, and reduced resting RF thickness emerged as an independent associated factor for slower walking speed in patients with KOA. Strengthening of periarticular muscles may therefore reduce the risk of further joint degeneration (39).

Decreased KE strength is a well-documented feature of KOA progression, accompanied by structural alterations-namely reduced muscle thickness and CSA (39). Moreover, a bidirectional two-sample Mendelian randomization study suggested that low muscle mass and reduced strength increase KOA risk and progression, while pain and limited mobility further accelerate muscle loss, creating a vicious cycle (40). Although our cross-sectional data are consistent with this framework, we cannot establish causality. Nonetheless, the observed associations suggest that interventions targeting pain relief and muscle structure merit further investigation.

### Dynamic tibial rotation as an independent biomechanical marker

We employed a 3D gait analysis system (Noraxon, a portable wireless wearable sensor system) to assess dynamic tibial rotation during walking. Compared with healthy controls, KOA patients exhibited significantly reduced total tibial rotation (16.6° ± 3.5° vs. 20.1° ± 2.9°, *p* < 0.001, Cohen's *d* = −1.067), representing a mean reduction of approximately 3.5°in rotational excursion during the gait cycle. Importantly, reduced total tibial rotation remained independently associated with slower gait speed (Beta = 0.194, *p* = 0.027) even after adjusting for pain and RF thickness. This finding indicates that tibial rotation provides incremental biomechanical information beyond conventional clinical evaluation—a patient with well-controlled pain and preserved quadriceps thickness may still exhibit clinically significant rotational deficits that impair mobility, a distinction with direct therapeutic implications. While full 3D motion capture is not yet routine in geriatric practice, the wearable system used here requires minimal space and technician training, making it more accessible than fixed-laboratory systems.

Previous studies in healthy individuals have shown that mild physiological external rotation optimizes patellar tracking and distributes joint contact stresses, mitigating cartilage and meniscal wear (41–43). In contrast, our KOA patients exhibited worse dynamic tibial internal and external rotation during walking. Abnormal rotational kinematics-whether external or internal-alter contact mechanics in the patellofemoral and tibiofemoral joints and substantially worsen mediolateral load imbalance (44). This aberrant shear stress can accelerate cartilage damage, compromise lower-limb alignment, and impair meniscal function (45).

### Pain and its associations

In univariate analyses, pain severity (VAS score) was negatively correlated with both tibial internal rotation (*r* = −0.208, *p* = 0.023) and total knee rotation (*r* = −0.217, *p* = 0.017) in patients with KOA, indicating that greater pain was associated with reduced rotational excursion. Such abnormal stress concentration may induce tibial deformities (e.g., altered hip-knee-ankle angle) and promote OA progression (46, 47). However, in the multivariate regression model (Table 5), only higher BMI (Beta = 0.235, *p* = 0.008), female sex (Beta = 0.237, *p* = 0.008), and slower gait speed (Beta = −0.286, *p* = 0.001) remained independently associated with pain severity, while tibial rotation did not enter the final model. This suggests that while pain and tibial rotation are correlated at the univariate level, their association is not independent of other clinical factors. The co-occurrence of reduced rotational excursion and greater pain may reflect shared pathways involving joint instability and altered loading, which together contribute to abnormal stress distribution and potentially influence disease progression over time.

Consistent with a meta-analysis of cohort and case-control studies, female sex and BMI were significantly correlated with pain in our KOA patients (8). Pain was negatively associated with walking speed, whereas muscle strength showed a positive association. These findings support a vicious-cycle model in which pain reduces walking speed, slower gait restricts daily activity, and consequent muscle wasting may, in turn, intensify pain.

### Knee ROM and proprioception

Patients with KOA also displayed worse knee flexion ROM and joint position sense (proprioception) than healthy controls, and both measures were significantly associated with walking speed in both groups. In KOA, degradation of the mechanoreceptors blunts proprioceptive feedback, leading to joint instability, increased risk of falls, and greater disability (48). Prior rehabilitation studies have shown that enhanced proprioceptive input improves overall knee motor control and mobility (49, 50). In the healthy control group, older age, reduced knee flexion ROM, poorer position sense, and smaller RF thickness during contraction were identified as associated factors for walking speed, further underscoring the importance of these parameters across populations.

### Clinical and rehabilitation implications

Our findings carry clear clinical and rehabilitation implications. First, the independent association between reduced total tibial rotation and slower gait speed suggests that rehabilitation programs should incorporate kinematic retraining as a core component, alongside traditional quadriceps strengthening. Second, ultrasound-derived RF thickness at rest may serve as a practical, radiation-free screening tool in geriatric and primary care settings, particularly when dynamometry is not available, to help identify patients at risk of functional decline. Third, the independent link between pain and gait speed reinforces that pain management should be viewed not merely as symptomatic relief, but as a crucial intervention to preserve mobility and delay functional deterioration. Finally, the distinct sets of factors associated with pain vs. gait speed suggest the potential for phenotype-based rehabilitation, though this hypothesis requires prospective validation.

### Limitations

This study has several limitations. First, the single-center design and volunteer recruitment may limit generalizability and introduce selection bias. Second, the cross-sectional design precludes causal or temporal inference, so our findings should be interpreted as associational only. Third, several potential confounders—including standardized physical activity scale, medication use, comorbidities, limb alignment, depression, frailty, sarcopenia, and socioeconomic status—were unavailable, which may have resulted in residual confounding. Fourth, the predominantly female sample (85% in KOA, 82.4% in controls), while consistent with KOA epidemiology, limits generalizability to males; sex was associated with pain but not gait speed, yet the small male subgroup (*n* = 18) in KOA precluded stratified analyses. Fifth, the absence of radiographic data precluded Kellgren-Lawrence grading determination for some KOA patients and asymptomatic KOA screening in controls who refused X-ray due to concerns about radiation exposure. Sixth, given the exploratory nature of some analyses, we did not apply corrections for multiple comparisons, which may inflate type I error; thus, these findings require validation in future studies. Seventh, we did not explore interaction effects among the independent variables due to sample size constraints. Such analyses would have been underpowered and prone to overfitting. Finally, as an observational study, no rehabilitation intervention was implemented. Future prospective studies with comprehensive confounder assessment, balanced sex representation, and radiographic evaluation are warranted to confirm our findings.

## Conclusion

In conclusion, our study provides findings that older adults with KOA exhibit multidimensional impairments-including reduced gait speed, limited ROM, proprioceptive deficits, altered RF morphology, and abnormal tibial rotation-each contributing to the clinical presentation. The independent associations of RF thickness and tibial rotation with gait speed, and of BMI, sex, and gait speed with pain, highlight the need for comprehensive, mechanism-based assessments. Future longitudinal studies are warranted to clarify causal pathways and to evaluate whether interventions targeting muscle structure and rotational kinematics can improve mobility and reduce pain in this population.

## Data Availability

The original contributions presented in the study are included in the article/supplementary material, further inquiries can be directed to the corresponding author.
